# Using mitochondrial DNA as a biosensor of early cancer development

**DOI:** 10.1038/sj.bjc.6602711

**Published:** 2005-08-03

**Authors:** M A Birch-Machin

**Affiliations:** 1 Dermatological Sciences, Medical School, University of Newcastle, Newcastle upon Tyne NE2 4HH, UK

Mitochondria can perform multiple cellular functions including energy production, cell proliferation and apoptosis. Each human cell contains hundreds to several thousand copies of the 16.5 kb human mitochondrial genome, which incidentally exhibits a maternal pattern of inheritance. This closed circular genome encodes 13 polypeptides of the respiratory chain complexes, as well as 22 transfer RNAs and two ribosomal RNAs used in mitochondrial protein synthesis. Compared to nuclear DNA, mitochondrial DNA (mtDNA) is highly susceptible to damage because it is not associated with protective histones, it is continually exposed to high levels of reactive oxygen species (ROS) generated by oxidative phosphorylation, and there is a limited capacity for mtDNA repair. The complete mtDNA sequence was determined in 1981 and resequenced in 1999. A growing collection of reported mtDNA mutations and rearrangements has been associated with muscle and neurodegenerative diseases (Birch-Machin, 2000).

## MTDNA DAMAGE AND CANCER

Mitochondria have been implicated in the carcinogenic process because of their role in apoptosis and other aspects of tumour biology. In particular, study of mtDNA mutations in cancer is a rapidly expanding area that explores the links of neoplastic growth with DNA modifications within this organelle. Many types of human malignancy such as colorectal, liver, breast, lung, prostate, skin and bladder cancer have been shown to harbour somatic mtDNA mutations (Copeland *et al*, 2002; Durham *et al*, 2003; Petros *et al*, 2005). In this issue of the BJC, Nishikawa and colleagues from Hyogo Medical School, Japan, present their findings that indicate accumulation of mtDNA mutations with colorectal carcinogenesis in ulcerative colitis (UC). In addition, the levels of 8-OHdG, a DNA adduct produced by ROS were significantly higher in UC than in control. Taking both observations together, the authors postulate the interesting idea that the high incidence of mtDNA mutations is enhanced in the mucosal cells of the patients with UC by a process of oxidative stress caused by the chronic inflammation. This in turn means that malignant transformation occurs more easily than in normal subjects. There are a number of important aspects surrounding this article some of which deserve wider reflection.

## MTDNA AS A SENSITIVE BIOSENSOR OF GENETIC DAMAGE

There are many mitochondrial genomes (2–10 copies) per mitochondria and many mitochondria per cell (a mammalian cell typically contains 200–2000 mitochondria). As a consequence, therefore, mitochondrial genomes can tolerate very high levels (up to 90%) of damaged DNA through complementation by the remaining wild type. This fact coupled with the limited DNA repair capacity of mtDNA can lead to the accumulation of genetic damage without compromising cell function, that is, two factors which are necessary requirements for a reliable and sensitive biosensor.

## ROS IN CANCER CELLS

Growing evidence suggests that cancer cells exhibit increased intrinsic ROS stress, due, in part, to oncogenic stimulation, increased metabolic activity and mitochondrial malfunction. Since the mitochondrial respiratory chain is a major source of ROS generation in the cells and the naked mtDNA molecule is in close proximity to the source of ROS, the vulnerability of the mtDNA to ROS-mediated damage appears to be a mechanism to amplify ROS stressing cancer cells (Pelicano *et al*, 2004).

## PROTEOMIC ANALYSIS OF CANCER-CELL MITOCHONDRIA

Despite the increased identification of signatures of mtDNA damage in transformed cells, the phenotypic effects of these genetic changes remain to be established. Research into the identification of altered expression patterns of mitochondrial proteins in cancer cells has been made possible by the relatively recent development of mitochondrial functional proteomics. The potential of this field may be realized in the identification of new markers and risk assessment as well as therapeutic targets. However, proteomic analyses face more challenges than the genomic approach. For example, the behaviour of proteins is largely determined by the tertiary structure of the molecule which puts constraints on affinity-based assays to capture protein targets. In addition, many proteins exist at very low levels, which in the absence of an amplification system equivalent to the polymerase chain reaction for DNA would make them difficult to identify and subsequently analyse. Finally, protein function is not necessarily correlated to quantity but also by rates of synthesis and degradation, reversible modification and RNA splicing (Verma *et al*, 2003).

## FUTURE APPROACHES

Despite the difficulties with mitochondrial proteomics, it is likely that the combination of the mitochondrial genetic and the proteomic approaches will provide an effective double edge sword in the fight against cancer. It is hoped that this strategy will provide specific genetic markers and protein profiles which will provide early detection, risk assessment and new targets for treatment. It may also help to answer the much debated question as to whether the observed mtDNA damage has a primary and causative link to the process of cancer development or it may simply represent a secondary bystander effect, which reflects an underlying nuclear DNA instability. One cautionary note in all these studies is the use of appropriate control tissue for cancer studies. This is highlighted by the work of Durham *et al*., who found that the traditional use of histologically ‘normal’ perilesional skin in nonmelanoma skin cancer studies had several limitations since the ‘normal tissue’ also harbored UV-induced mtDNA damage. These results may suggest, however, that neoplasia is evident at the molecular level before pathohistological changes are visible, a feature which itself may provide a powerful biosensor of early cancer development.
